# Post-Traumatic Sepsis Is Associated with Increased C5a and Decreased TAFI Levels

**DOI:** 10.3390/jcm9041230

**Published:** 2020-04-24

**Authors:** Jan Tilmann Vollrath, Ingo Marzi, Anna Herminghaus, Thomas Lustenberger, Borna Relja

**Affiliations:** 1 Department of Trauma, Hand and Reconstructive Surgery, Goethe University, 60590 Frankfurt, Germany; Tilmann.Vollrath@kgu.de (J.T.V.); Ingo.Marzi@kgu.de (I.M.); Thomas.Lustenberger@kgu.de (T.L.); 2 Department of Anesthesiology, Duesseldorf University Hospital, 40225 Duesseldorf, Germany; Anna.Herminghaus@med.uni-duesseldorf.de; 3 Experimental Radiology, Department of Radiology and Nuclear Medicine, Otto von Guericke University, 39120 Magdeburg, Germany

**Keywords:** polytrauma, sepsis, complement, patients

## Abstract

Background: Sepsis frequently occurs after major trauma and is closely associated with dysregulations in the inflammatory/complement and coagulation system. Thrombin-activatable fibrinolysis inhibitor (TAFI) plays a dual role as an anti-fibrinolytic and anti-inflammatory factor by downregulating complement anaphylatoxin C5a. The purpose of this study was to investigate the association between TAFI and C5a levels and the development of post-traumatic sepsis. Furthermore, the predictive potential of both TAFI and C5a to indicate sepsis occurrence in polytraumatized patients was assessed. Methods: Upon admission to the emergency department (ED) and daily for the subsequent ten days, circulating levels of TAFI and C5a were determined in 48 severely injured trauma patients (injury severity score (ISS) ≥ 16). Frequency matching according to the ISS in septic vs. non-septic patients was performed. Trauma and physiologic characteristics, as well as outcomes, were assessed. Statistical correlation analyses and cut-off values for predicting sepsis were calculated. Results: Fourteen patients developed sepsis, while 34 patients did not show any signs of sepsis (no sepsis). Overall injury severity, as well as demographic parameters, were comparable between both groups (ISS: 25.78 ± 2.36 no sepsis vs. 23.46 ± 2.79 sepsis). Septic patients had significantly increased C5a levels (21.62 ± 3.14 vs. 13.40 ± 1.29 ng/mL; *p* < 0.05) and reduced TAFI levels upon admission to the ED (40,951 ± 5637 vs. 61,865 ± 4370 ng/mL; *p* < 0.05) compared to the no sepsis group. Negative correlations between TAFI and C5a (*p* = 0.0104) and TAFI and lactate (*p* = 0.0423) and positive correlations between C5a and lactate (*p* = 0.0173), as well as C5a and the respiratory rate (*p* = 0.0266), were found. In addition, correlation analyses of both TAFI and C5a with the sequential (sepsis-related) organ failure assessment (SOFA) score have confirmed their potential as early sepsis biomarkers. Cut-off values for predicting sepsis were 54,857 ng/mL for TAFI with an area under the curve (AUC) of 0.7550 (*p* = 0.032) and 17 ng/mL for C5a with an AUC of 0.7286 (*p* = 0.034). Conclusion: The development of sepsis is associated with early decreased TAFI and increased C5a levels after major trauma. Both elevated C5a and decreased TAFI may serve as promising predictive factors for the development of sepsis after polytrauma.

## 1. Introduction

Trauma is responsible for one out of 10 deaths worldwide and is among the most frequent causes of mortality, notably among young people [1]. Traumatized patients who survive the initial post-injury phase are at high risk to die from late-occurring complications such as multiple organ failure (MOF), respiratory complications, and/or sepsis [2,3,4,5]. During sepsis, the activation of both coagulation and complement systems, which can be triggered by a pathogen itself or damaged tissues, is essential as a reaction to tissue injury and for an adequate host defense against pathogens [6,7]. Nevertheless, abnormalities in the well-regulated interactions between these systems or their excessive activation can be harmful and lead to thrombosis or disseminated intravascular coagulation [6,7].

Thrombin-activatable fibrinolysis inhibitor (TAFI), a procarboxypeptidase 2 or carboxypeptidase U, is a carboxypeptidase B-like proenzyme with a molecular weight of 56 kDa [8]. Upon activation by the thrombin-thrombomodulin complex at the vascular endothelial surface, its activated TAFI form (TAFIa) inhibits fibrinolysis by removing C-terminal lysines from fibrin, which are imperative for the efficient formation of plasmin [8,9]. In a septic rat model with *Pseudomonas aeruginosa*, the inhibition of TAFIa reduced the systemic inflammatory response, thus suggesting that TAFI plays an important role in the deterioration of organ dysfunction in sepsis [10]. In a rat model of a sepsis-complicating burn injury, TAFI was elevated after 24 and 72 h, confirming the close link between inflammation and coagulation [11]. Septic patients, as well as healthy study subjects with low-grade lipopolysaccharide (LPS)-induced endotoxemia, showed decreased levels of TAFI [12,13], and in polytraumatized patients, TAFI levels inversely correlated with the inflammation-associated development of complications [14].

Anaphylatoxin C5a is a strong chemoattractant signal that is involved in the regulation of the innate immune system, playing a key role in host homeostasis, inflammation, and defense against pathogens [15,16,17,18,19]. Upon activation through the cleavage of C5 to C5a and C5b by the C5-convertase, C5a takes part in the recruitment and activation of inflammatory cells like neutrophils, eosinophils, T lymphocytes, and monocytes [19,20]. Recently, in a baboon model of *Escherichia coli* bacteremia, it was shown that complement-mediated bacteriolysis had a detrimental effect by inducing a release of LPS and fulminant inflammation [21]. The inhibition of C5 cleavage blocked sepsis-induced inflammation, decreased the associated consumptive coagulopathy, and protected organ functions, resulting in improved survival [21].

Besides its role in coagulation, TAFI has been shown to have anti-inflammatory properties, thus being able to inactivate activated complement factors C3a and C5a [22,23]. Therefore, in the present study, we included severely injured trauma patients with sepsis to determine whether TAFI might represent a possible link between inflammation/complement and coagulation in sepsis.

## 2. Materials and Methods

### 2.1. Ethics

This study was performed at the University Hospital of the Goethe University Frankfurt with the institutional ethical approval in accordance with the Declaration of Helsinki and following the Strengthening the Reporting of Observational Studies in Epidemiology (STROBE) guidelines (167/05). Written informed consent was obtained from all enrolled patients in accordance with ethical standards. All patients signed the informed consent forms themselves, or informed consent was obtained from the nominated legally authorized representative consenting on the behalf of participants, as approved by the ethical committee.

### 2.2. Patients

Patients were included according to the following criteria: history of penetrating or blunt trauma with an injury severity score (ISS) ≥ 16 and between 18 and 80 years of age. Patients with pre-existing immunological disorders, concomitant acute myocardial infarction, immunosuppressive or anticoagulant medication, burns, thromboembolic events, and/or lethal injury were excluded. All patients were treated according to the Advanced Trauma Life Support (ATLS^®^) standards and the polytrauma guidelines. While haemodynamically instable patients received immediate surgery, haemodynamically stable patient underwent whole-body computed tomography. Upon arrival to the emergency room, the following demographic and clinical data were collected: age; gender; blood pressure; respiratory rate; heart rate; temperature; mechanism of injury; abbreviated injury scale for each body region (head, chest, abdomen, and extremity); and the general injury severity (ISS), as described before [24]. Routine blood gas analysis (including pH and lactate) was performed upon admission to the hospital. The numbers of fresh frozen plasma (FFP) and packed red blood cell (PRBC) units transfused within the first 24 h and during further clinical course were recorded. The diagnose of sepsis was assessed by both the 2005 criteria outlined by the International Sepsis Forum [25], as well as by the revised definition criteria according to the Sepsis-3 criteria [26,27]. Systemic inflammatory response syndrome (SIRS) was defined by fulfilling at least two of the following criteria: heart rate > 90 beats per minute, respiratory rate > 20 per minute or arterial carbon dioxide tension (PaCO_2_) < 32 mm Hg, body temperature >38 °C or <36 °C, and white blood cell count >12,000 cells/mm^3^ or <4000 cells/mm^3^ or with >10% immature forms. According to the old definition, sepsis was diagnosed when the patients fulfilled criteria for SIRS and had evidence for an infection. However, limitations of that definition, including an excessive focus on inflammation and the inadequate specificity and sensitivity of the SIRS criteria, led to the Sepsis-3 definition, in which sepsis was defined as life-threatening organ dysfunction caused by a dysregulated host response to infection. The authors have summarized that, for clinical operationalization, organ dysfunction can be represented by an increase in the sequential (sepsis-related) organ failure assessment (SOFA) score of 2 points or more, which is associated with an enhanced in-hospital mortality risk [26,27]. Medical records were analyzed regarding length of in-hospital stay, length of ICU stay, and in-hospital mortality.

### 2.3. Blood Processing and Analysis

Blood samples were obtained from 48 severely traumatized patients as early as possible after admission to the emergency department (ED) and until day ten daily in pre-chilled ethylene-diaminetetraacetic acid (EDTA) or heparin vacuum tubes (BD vacutainer; Becton Dickinson Diagnostics, Aalst, Belgium) for routine diagnostics or for laboratory investigations and were kept on ice until centrifugation at 2000× *g* for 15 min at 4 °C. Afterwards, supernatants were stored at −80 °C until analysis. The mean time between injury and acquisition of the blood sample was 81 ± 8 min.

TAFI levels were measured by using a commercially available ELISA (IMUCLONE™ TAFI ELISA; American Diagnostica Inc., Stamford, CT, USA) according to the manufacturer’s instructions. The TAFI antigen level is expressed as a percentage of normal pooled plasma. C5a levels were determined using a commercially available Human C5a ELISA kit II (BD Biosciences, Heidelberg, Germany) according to the manufacturer’s instructions. Blood counts were measured using the Sysmex XE-2100 automated blood cell counter (Sysmex Europe GmbH, Norderstedt, Germany). pH, lactate, and PaCO_2_ were determined using a standard clinical blood gas analysis device (ABL800 Flex, Radiometer, Krefeld, Germany).

### 2.4. Statistics

All data were tested for normal distribution by a Kolmogorov-Smirnov-Lillieford’s test. Continuous variables were compared using the Mann-Whitney U test, and categorical variables were analyzed with the two-sided Fisher´s exact test. Data are presented as the mean ± standard error of the mean (SEM) unless otherwise stated. Receiver-operator curves (ROC) were calculated to analyze the optimal cut-off values. The sample size was performed based on the mean TAFI data obtained from the pilot study in Relja et al. [14]. Based on the mean values, a Cohen‘s d of 1.060 and an effect size of *r* = 0.468 were calculated. Applying a power of 0.8, a group size of 15 per group is necessary to reach significant results in an unpaired *t*-test (α = 0.05). Results were considered as statistically significant when the *p*-value was <0.05. Statistical analysis was performed using GraphPad Prism 5.0 software (GraphPad Software Inc., San Diego, CA, USA).

## 3. Results

### Main Findings

A cohort of 48 patients (33 male) met the inclusion criteria and were included in the study. Mean patient age was 52.23 ± 2.74 years, and all patients were substantially injured with a mean ISS of 24.95 ± 1.75. Of these, 14 patients with an ISS of 23.36 ± 2.79 developed sepsis during the clinical course, while 34 patients with an ISS of 25.78 ± 2.36 did not develop sepsis or septic complications. Table 1 summarizes the general patient and injury characteristics of the study population. There were no significant differences between the sepsis and no sepsis group with regard to the general patient and injury characteristics. The mean time between the onset of trauma and arrival to the ED was 66.17 ± 4.85 min. The mean time until admission to the ED between the cohort of septic patients vs. not septic patients did not statistically differ (65.40 ± 11.55 vs. 66.50 ± 5.17 min).

To investigate the influence of blood transfusions concerning the development of sepsis after polytrauma, the number of fresh frozen plasma (FFP) and packed red blood cells (PRBC) during the first 24 h after admittance to the emergency department and during the whole clinical course were determined. Patients belonging to the sepsis group received significantly more units of packed red blood cells during the whole clinical treatment compared to the no sepsis group (10.50 ± 2.50 vs. 5.46 ± 21.74; *p* < 0.05). As depicted in Table 2, there were no significant differences between these two groups concerning the units of packed red blood cells during the first 24 h, the systolic blood pressure <90 mm Hg at admittance to the ED, the number of transfused units of fresh frozen plasma within 24 h or during the whole clinical course, or the body temperature at admittance to the ED. Furthermore, no significant differences among the groups in the international normalized ratio (INR), activated partial thromboplastin time (PTT), or platelet counts (PLT) were assessed.

A significantly prolonged stay in the intensive care unit was observed in septic patients (22.14 ± 4.12 days vs. 7.00 ± 1.16; *p* < 0.0001), and in-hospital stays lasted significantly longer for this group (41.07 ± 6.43 vs. 19 ± 2.90; *p* < 0.001) compared to the group without septic complications. A total of six patients (12.5%) died, with a significantly higher number of patients in the sepsis group compared with the no sepsis group (four vs. two; *p* < 0.05; Table 3).

**Figure 1:**
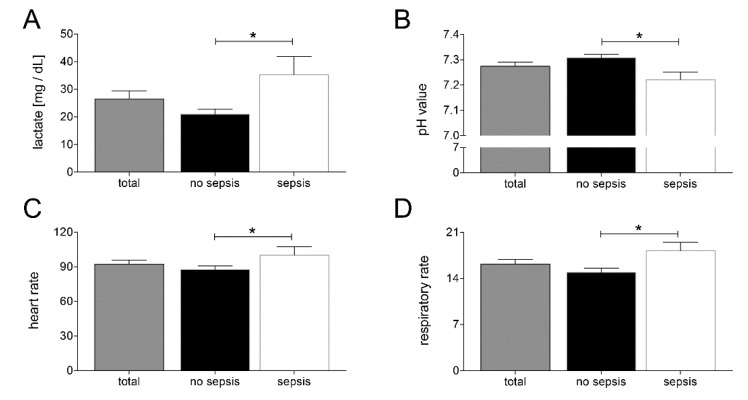
Upon admission of severely injured trauma patients (*n* = 48) to the emergency department, lactate levels (**A**), pH values (**B**), heart rate (**C**), and respiratory rate (**D**) were measured. Patients were stratified to those who developed sepsis in their clinical course (sepsis, *n* = 14) vs. those who did not develop infectious complications (no sepsis, *n* = 34). Data are shown as mean ± SEM. * *p* < 0.05 sepsis vs. no sepsis.

In order to further investigate the differences of clinical features at admission to the ED between patients developing sepsis during the clinical course and patients who did not develop sepsis, we compared lactate concentrations and pH values in the blood, heart rate, and respiratory rate in our study cohort. As shown in Figure 1, patients who developed sepsis had significantly higher amounts of lactate in their blood and a more acidic constellation of their acid bases balance, as depicted by significantly lower pH values (*p* < 0.05; Figure 1A,B). Furthermore, patients who developed sepsis during the clinical course had significant changes of their vital parameters, as they had higher respiratory and heart rates at their admission to the ED (*p* < 0.05; Figure 1C,D).

**Figure 2:**
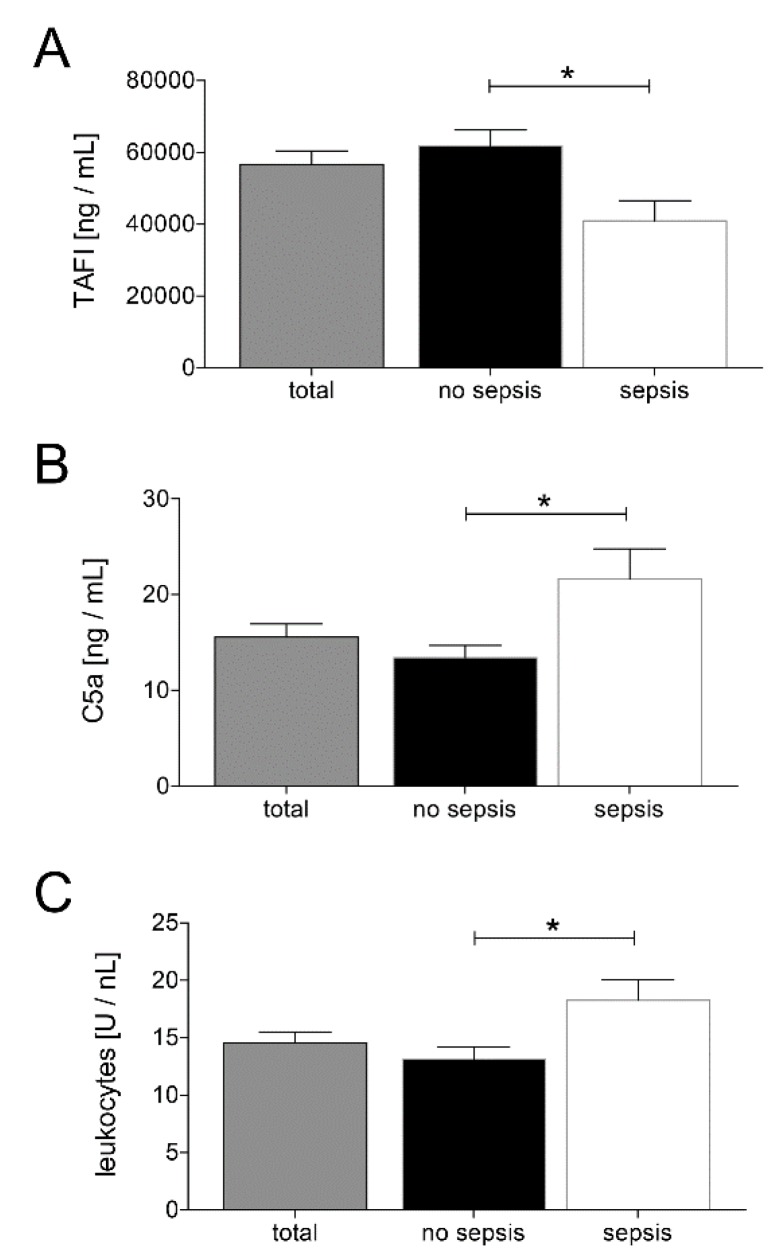
Upon admission of severely injured trauma patients (*n* = 48) to the emergency department, plasma thrombin-activatable fibrinolysis inhibitor (TAFI) levels (**A**), plasma C5a levels (**B**), and leukocyte counts (**C**) were determined. Patients were stratified to those who developed sepsis in their clinical course (sepsis, *n* = 14) vs. those who did not develop infectious complications (no sepsis, *n* = 34). Data are shown as mean ± SEM. * *p* < 0.05 sepsis vs. no sepsis.

In order to investigate changes in coagulation and inflammation, we measured TAFI and C5a levels at admission to the ED. Patients belonging to the sepsis group had significantly lower TAFI levels and significantly higher C5a levels compared to the no sepsis group, as shown in Figure 2A,B, respectively (*p* < 0.05). Moreover, patients from the sepsis group showed significantly increased leukocytes counts compared with the no sepsis group (*p* < 0.05; Figure 2C).

**Figure 3:**
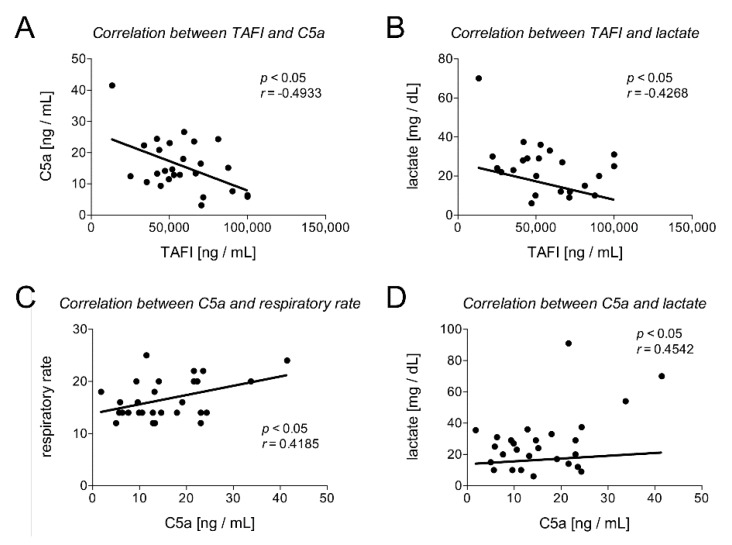
Correlation analysis between TAFI and C5a (**A**), TAFI and lactate (**B**), C5a and respiratory rate (**C**), and C5a and lactate (**D**) are shown. *r*: Pearson coefficient.

In addition, we analyzed correlations between TAFI and/or C5a and clinical (heart and respiratory rate) or laboratory parameters (pH value, leukocyte count, lactate value, INR, thromboplastin time (TPT), PTT, and PLT) and the SOFA score. TAFI and C5a, as well as TAFI and lactate, have shown a significant negative correlation (TAFI and C5a: Pearson *r* = −0.4933, *p* < 0.0104 and TAFI and lactate: Pearson *r* = −0.4268, *p* < 0.0423; Table 4 and Figure 3A,B). Furthermore, C5a and lactate, as well as C5a and the respiratory rate, had a significant correlation (C5a and lactate: Pearson *r* = 0.4542, *p* < 0.0173 and C5a and respiratory rate: Pearson *r* = 0.4185, *p* < 0.0266; Table 4 and Figure 3C,D). In addition, correlation analysis of TAFI and C5a with the SOFA score have shown a significant negative correlation of TAFI with the SOFA score (Pearson *r* = −0.3865, *p* = 0.0422) and a significant positive correlation of C5a with the SOFA score (Pearson *r* = 0.3795, *p* = 0.0386) (Table 4).

**Figure 4:**
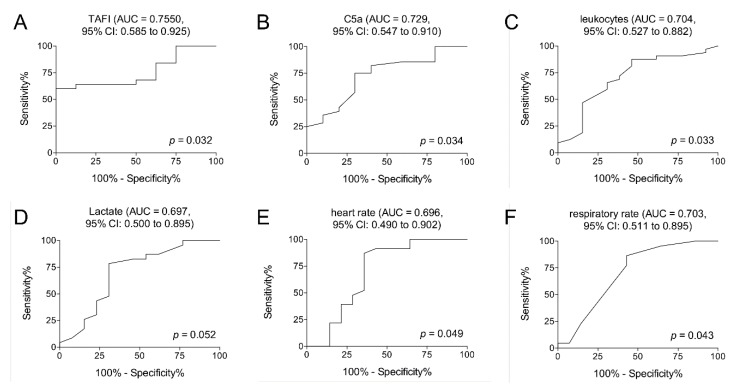
Receiver operating curve analyses on TAFI (**A**), C5a (**B**), leukocyte counts (**C**), lactate levels (**D**), heart rate (**E**), and respiratory rate (**F**) at admission to the emergency department to predict the development of sepsis after polytrauma are demonstrated. *p* < 0.05: statistically significant, AUC: area under the curve, and CI: confidence interval.

To investigate which of the examined parameters in this study (TAFI, C5a, leukocytes count, lactate, heart rate, and respiratory rate) could provide a prognostic marker for the development of sepsis after polytrauma, the predictive power of each parameter has been calculated using ROC analyses. While the ROC analysis for lactate did not show a statistically significant area under the ROC curve (*p* = 0.052), TAFI showed an optimal cut-off for predicting sepsis on admission to the ED at 54,857 ng/mL, with a sensitivity of 64% and specificity of 87.50%. The AUC was 0.7550 (95% CI: 0.585 to 0.925; *p* < 0.032; Table 5 and Figure 4A). C5a showed an optimal cut-off at 17 ng/mL on admission to the ED for predicting sepsis in the clinical course, with a sensitivity of 75.00%, specificity of 70.00%, and an AUC of 0.7286 (95% CI: 0.547 to 0.910; *p* < 0.034; Table 5 and Figure 4B). Leukocytes showed an optimal cut-off at 14.50 U/nl, with a sensitivity of 65.63%, specificity of 69.23%, and an AUC of 0.7043 (95% CI: 0.527 to 0.882; *p* < 0.033; Table 5 and Figure 4C). The heart rate showed an optimal cut-off at 102.5/min on admission to the ED for predicting sepsis in the clinical course, with a sensitivity of 86.96%, specificity of 64.29%, and an AUC of 0.6957 (95% CI: 0.490 to 0.902; *p* < 0.049; Table 5 and Figure 4E). The respiratory rate showed an optimal cut-off at 15/min, with a sensitivity of 63.64% and a specificity of 64.29%. The AUC was 0.7029 (95% CI: 0.511 to 0.895; *p* < 0.043; Table 5 and Figure 4F).

**Figure 5:**
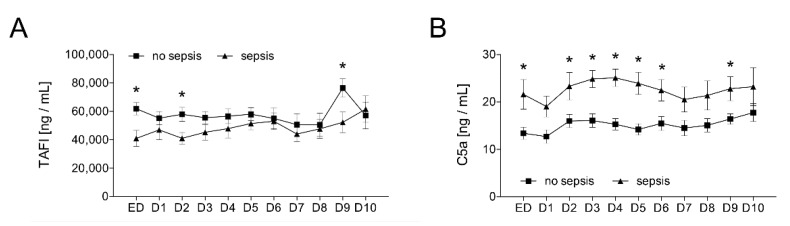
Upon admission of severely injured trauma patients (*n* = 48) to the emergency department (ED), plasma TAFI levels (**A**) and plasma C5a levels (**B**) were determined daily for ten days (D1–D10). Patients were stratified to those who developed sepsis in their clinical course (sepsis, *n* = 14) vs. those who did not develop infectious complications (no sepsis, *n* = 34). Data are shown as mean ± SEM. * *p* < 0.05 sepsis vs. no sepsis at the indicated time.

The results in Figure 5 reflect the dynamic course of both parameters after trauma and sepsis. The distribution of the TAFI and C5a values in the samples obtained in the ED and subsequent daily measurements for ten consecutive days show that the TAFI was significantly decreased on admission, day 2, and on day 9 in the sepsis group compared to the control group without sepsis (*p* < 0.05; Figure 5A). On the contrary, the C5a values were significantly increased on admission, days 2–6, and on day 9 in the sepsis group compared to the control group without sepsis (*p* < 0.05; Figure 5B).

## 4. Discussion

Polytraumatized patients who survive the initial post-injury phase are at a serious risk to die from septic complications during their further clinical treatment [18]. Both the inflammatory/complement and the coagulation systems contribute to these harmful events. Regarding this, it is important to note that the cross-talk between the inflammation/complement and the coagulation occurs in both directions, as coagulation can affect the inflammatory activity as well as inflammation is able to activate coagulation [14,28,29]. TAFI is a carboxypeptidase B-like proenzyme, which, on the one hand, upon its activation to TAFIa by the thrombin-thrombomodulin complex inhibits fibrinolysis by removing C-terminal lysines from fibrin [8,9]. On the other hand, activated complement factors C3a and C5a can be inactivated by TAFIa [22,30]. The data suggests that TAFI may be a potential link between the inflammation/complement and the coagulation systems.

In the underlying study, we observed significantly reduced TAFI levels in patients developing sepsis during their clinical course after polytrauma compared to polytraumatized patients without septic complications. This difference occurred early upon admission of the patients to the ED. In contrast, C5a levels were significantly increased in septic patients after polytrauma. A significant negative correlation between TAFI and C5a levels was found. In addition, the correlation analyses of TAFI and C5a with the SOFA score have demonstrated a significant negative correlation of TAFI and a significant positive correlation of C5a with the SOFA score. These findings enhance markedly the impact of both TAFI and C5a as biomarkers, which may indicate later-occurring development of the post-traumatic sepsis. However, whether reduced TAFI levels in septic patients after polytrauma derive from, e.g., consumption in the course of coagulopathy or whether initially reduced TAFI levels potentially put patients at risk for septic complications still remains elusive. It can be speculated that maybe upon reduced TAFI levels, missing inactivation of C3a and C5a may lead to an immunological imbalance and an excessive immunological reaction. As nicely reviewed and originally published by the expert group around Huber-Lang, anaphylatoxin C5a appears in the circulation of humans within 20 min post-polytrauma, and its levels have been related to the mortality rate [31,32]. That data indicated an almost synchronical rapid activation and dysfunction of the complement, suggesting a trauma-induced complementopathy early after injury [32]. Furthermore, the authors suggest that these events may participate in the impairment of the post-traumatic innate immune response. Within the time latency between 20 min of the complement activation, as shown by Huber-Lang, and 66 min until admission of patients to the ED, as shown in our study, certainly, a trauma-induced complementopathy may have occurred. Thus, levels of both TAFI and C5a may have been different at the onset of trauma, and we cannot exclude any alterations which may have emerged until the blood sampling in the ED was performed. Thus, the latency between the timepoint of trauma and sampling in the ED must be taken into account when interpreting the results. Relja et al. reported elevated TAFI levels in polytraumatized patients with and without infectious complications but with a significantly higher increase in both TAFI and TAFIa in patients without infectious complications [14]. This supports the hypothesis that reduced TAFI levels may increase the risk for the development of septic complications. In line with these findings, Zeerleder et al. as well reported significantly decreased TAFI levels in septic patients compared to healthy controls [33]. Interestingly, in our pilot study, we found that TAFI levels at admission to the ED and at day 4 after the trauma were significantly lower in the smaller cohort of patients with complications compared to the control group [14]. In that study, we were able to demonstrate that TAFI levels inversely correlated with the inflammation-associated development of not-further-defined complications after major trauma. Furthermore, coagulopathic patients, however, experienced significantly lower levels of activated TAFI on admission and on days six to eight [34]. Based on those results, the finding that TAFI and C5a levels immediately at admission to the ED can stratify patients who are going to develop sepsis in their clinical course is certainly a promising result in terms of a search for early sepsis biomarkers. Yet, it remains to be noted that, in our study, we did not prospectively evaluate the levels of both potential biomarkers and that this remains to be elucidated in further studies.

Naito et al. investigated the link between C5a and TAFI with regard to acute lung injury and demonstrated that, in TAFI-deficient mice, LPS treatment induced a more severe acute lung injury (ALI) compared to wild-type mice [35]. Furthermore, the authors observed significantly higher concentrations of IL-1beta, TNF-beta, and IL-6, along with increased lung infiltration of neutrophils, in TAFI-deficient mice [35]. Naito et al. concluded that TAFI plays a critical role in the pathogenesis of ALI via its ability to regulate the activity of complement factor C5a [35]. Nishimura et al. reported worse alveolitis in TAFI knock-out mice than in wild-type (WT) mice with C5a instillation directly into the lungs [36]. In line with these results, we observed decreased TAFI levels and elevated C5a levels in patients who developed septic complications after polytrauma. More importantly, TAFI correlated negatively with C5a. Fujiwara et al. investigated the role of TAFI in allergic bronchial asthma and showed significantly worse airway responsiveness and increased pulmonary concentrations of IL-5 and osteopontin in a TAFI-deficient mouse model [37]. Pretreatment with a C5a antibody significantly attenuated these effects, and thus, the authors concluded that TAFI plays a protective role in the pathogenesis of allergic inflammation, probably by inhibiting the complement system [37]. In a model of inflammatory arthritis (anticollagen antibody–induced arthritis (CAIA)), Song et al. investigated the role of TAFI and showed that TAFI deficiency exacerbated inflammatory arthritis and, furthermore, that the cleavage of C5a by TAFI suppressed the ability of C5a to recruit immune cells to the affected joint [38]. Interestingly, the authors showed that the expression of both TAFI and C5a in synovial fluid was higher in patients with autoimmune arthritis than in those with osteoarthritis and concluded that TAFI plays an important role in attenuating local C5a-mediated inflammation and, thus, represents a molecular link between inflammation and coagulation in autoimmune arthritis [38].

While the above-mentioned studies all unanimously reported that decreased/lower levels of TAFI led to aggravated disease severity, Mook-Kanamori et al. showed that higher protein levels of TAFI in cerebrospinal fluid were significantly associated with systemic complications in patients with bacterial meningitis [39]. The authors observed survival benefits in TAFI-deficient mice in a pneumococcal meningitis mouse model as compared to wild-type mice and concluded that, during the initial phase of infection, a low level or an absence of TAFI results in initially increased complement levels, which may improve survival (in the animal model) and reduce systemic complications in humans suffering from bacterial meningitis [39]. Taken together, it still remains elusive whether TAFI acts as an acute reactant marker that links homeostasis and inflammation or whether a lack of TAFI plays an active role in the development of septic complications after polytrauma.

Most clinical studies examining anticoagulant effects on thrombo-inflammation have been performed in the context of sepsis, although some specific examples relevant to ischemia/reperfusion injuries are also reported. Unfortunately, the data in septic patients in which inhibitors of thrombin have been applied, as well as their efficiency, are limited [40]. As an example, the overall benefit of low molecular weight heparin in patients with sepsis remains, despite considerable investigations, uncertain [40]. Although initial trials with the IV infusion to restore plasma ATIII levels were promising, larger studies did not provide any benefit for overall mortality, and consequently, Rhodes et al. have made specific recommendations against the use of ATIII in the updated International Guidelines for the Management of Sepsis and Septic Shock [40,41]. The use of other anticoagulant agents, e.g., recombinant human activated protein C, have failed to consistently demonstrate a reduction in 28-day all-cause mortality in clinical trials [42]. Currently, soluble recombinant human thrombomodulin (rhTM) is undergoing clinical evaluation for the treatment of severe sepsis [40]. ART-123, an rhTM, appears to be a safe intervention in critically ill sepsis patients in phase 2b trials [40,43].

Although this study has several limitations—of those, the most important, the limited sample size—it must be considered that there is no significant difference between demographic and trauma characteristics between evaluated groups, which certainly ensures better compatibility of assessed data. Nevertheless, it has to be taken into account that patients in the sepsis group received significantly more PRBC during the whole clinical course, while there was no significant difference within the first 24 h (Table 2). Sadjadi et al. reported that, in trauma patients, infection was associated with the transfusion of PRBC and that transfused patients had eight times the increased risk of infection independently of their injury severity [44]. Likewise, Claridge et al. reported a dose-dependent correlation between transfusions of PRBC and the development of infection in traumatized patients [45]. Based on the data of our study, we cannot fully exclude an effect of transfused PRBC either on TAFI or on C5a, and this remains to be elaborated in future works. Although several studies have reported that a PRBC transfusion can negatively affect clinical outcomes [46,47], it still remains unclear if TAFI is an acute phase reactant that links hemostasis and inflammation or whether it plays an active role in the development of post-traumatic sepsis. With regard to the effects of PRBC transfusions on, e.g., the complement, certain clinical syndromes such as transfusion-related acute lung injury (TRALI) and transfusion-associated circulatory overload (TACO) that occur within 6 h of blood transfusions indicate an association [48,49]. The major drivers of those complications and their pathogenesis, however, are complex and incompletely understood. The potential connection between the complement system and neutrophils is given, as well as the parallels between these syndromes and other acute pulmonary disorders, which are known for their involvement of the complement. Yet, similar to TAFI, we cannot exclude a direct impact of the PRBS transfusion on C5a levels or its activity; however, since the transfusion rates within the first 24 h after the trauma did not differ between the septic and nonseptic group, we believe that the data as demonstrated are plausible. Moreover, C5a levels remain increased in septic patients nearly throughout the complete observational period, and thus, we believe that this effect is associated with inflammatory changes caused by sepsis rather than with PRBC transfusions, which, anyway, were initially not significantly enhanced. However, the increased amount of totally transfused PRBC in our study must therefore be considered as a disturbing variable by interpreting the data. Further limitations of the study include the study setting, since this study was conducted in a single center. In addition, the heterogeneity of the traumatized patients, as well as the variable timing of measurements, must be carefully considered in data interpretation. Although there were sufficient measurements made later after the trauma to determine the roles of TAFI and C5a as indicators of sepsis, a long-term study also including septic patients from other pathologies is necessary, but this was not the aim of the study.

Early recognition and effective management of patients at risk for infectious complications are essential for improved outcomes. However, early recognition specifically of post-traumatic sepsis is impeded by the lack of clinically proven and utilized biomarkers. The current work in clinical settings has not yielded the requisite gold standards for the diagnosis and monitoring of post-traumatic sepsis. Yet, identifying patients at risk for the development of sepsis during their clinical course is of great importance for further clinical treatment. TAFI and C5a may be helpful, specifically in, e.g., decision-making about the timing for post-traumatic surgeries or indicate patients that should early receive antibiotic therapy or undergo further specific clinical tests of sepsis markers. With regard to complement factors or TAFI as potential biomarkers in general, clinical studies with large subject numbers are needed to obtain the complete temporal profiles of, e.g., activation of the complement factors or factor dynamics in the course of sepsis development. Presumably, this should provide a multipanel set of biomarkers, which can be coupled with routine lab tests and blood cultures for the diagnosis and monitoring of sepsis.

Taken together, in the underlying study, we were able to show that the development of sepsis after major trauma is associated with decreased TAFI levels and increased C5a levels. The significant negative correlation between TAFI and C5a supports the concept that reduced TAFI levels may lead to an exacerbation of septic complications and that TAFI might play a protective role by downregulating inflammation. Furthermore, irrespective of their possible pathomechanistical roles in the onset of sepsis, both TAFI and C5a might serve as very early diagnostic markers to detect patients at risk for the development of sepsis or septic complications.

## Figures and Tables

**Table 1:** Demographic and trauma characteristics of the study population. Data are given as mean ± standard error of the mean; *p* < 0.05. Abbreviations: a, no sepsis group; AIS, abbreviated injury scale; b, sepsis group; ISS, injury severity score; and ns, no significance.

Subjects and Trauma Characteristics	Total (*n* = 48)	No Sepsis ^a^ (*n* = 34)	Sepsis ^b^ (*n* = 14)	*p* < 0.05 a vs. b
**age (years), mean ± sem**	52.23 ± 2.74	48.45 ± 3.74	56,54 ± 3.68	ns
**gender (male, %)**	33 (68.75%)	22 (64.71%)	11 (78.57%)	ns
**trauma mechanism (falls)**	23 (47.92%)	16 (47.06%)	7 (50.00%)	ns
**ISS**	24.95 ± 1.75	25.78 ± 2.36	23.46 ± 2.79	ns
**AIS ≥ 3 (*n*, %)**				
**Head**	22 (45.83%)	16 (47.06%)	6 (42.86%)	ns
**Chest**	16 (33.33%)	11 (32.35%)	5 (35.71%)	ns
**Abdomen**	7 (14.58%)	4 (11.76%)	3 (21.43%)	ns
**Extremity**	10 (20.83%)	7 (20.59%)	3 (21.43%)	ns

**Table 2:** Physiologic characteristics of the study population. Data are given as mean ± standard error of the mean; *p* < 0.05. Abbreviations: a, no sepsis group; b, sepsis group; ED, emergency department; FFP, fresh frozen plasma; INR, international normalized ratio; ns, no significance; PLT, platelet; PRBC, packed red blood cells; PTT, activated partial thromboplastin time; and SBP, systolic blood pressure.

Physiologic Characteristics	Total (*n* = 48)	No Sepsis ^a^ (*n* = 34)	Sepsis ^b^ (*n* = 14)	*p* < 0.05 a vs. b
**SBP < 90 mm Hg (ED, *n*, %)**	5 (10.42%)	2 (5.71%)	3 (21.43%)	ns
**PRBC transfusion within 24 h (Units)**	4.84 ± 1.18	3.81 ± 1.53	7.14 ± 2.01	ns
**PRBC transfusion total (Units)**	6.98 ± 1.41	5.46 ± 21.74	10.50 ± 2.50	<0.05
**FFP transfusion within 24 h (Units)**	2.54 ± 0.69	1.65 ± 0.71	4.31 ± 1.45	ns
**FFP transfusion total (Units)**	2.68 ± 0.75	1.73 ± 0.75	4.43 ± 1.56	ns
**INR (ED)**	1.34 ± 0.08	1.36 ± 0.11	1.31 ± 0.06	ns
**PTT (ED, s)**	36.13 ± 2.79	37.12 ± 3.88	33.72 ± 1.62	ns
**PLT count (ED, × 10^3^/μL)**	201.40 ± 10.34	198.00 ± 12.44	210.30 ± 18.97	ns

**Table 3:** Outcome of the study population. Data are given as mean ± standard error of the mean; *p* < 0.05. Abbreviations: a, no sepsis group; b, sepsis group; ICU, intensive care unit.

Outcome	Total (*n* = 48)	No Sepsis ^a^ (*n* = 34)	Sepsis ^b^ (*n* = 14)	*p* < 0.05 a vs. b
**ICU stay (days)**	11.71 ± 1.82	7.00 ± 1.16	22.14 ± 4.12	<0.0001
**in-hospital stay (days)**	25.67 ± 3.13	19.00 ± 2.90	41.07 ± 6.43	<0.001
**in-hospital mortality (*n*, %)**	6 (12.5%)	2 (5.88%)	4 (28.57%)	<0.05

**Table 4:** Correlation analyses between thrombin-activatable fibrinolysis inhibitor (TAFI), C5a, leukocyte counts, lactate levels, pH values, heart and respiratory rates, sequential (sepsis-related) organ failure assessment (SOFA) score, international normalized ratio (INR), thromboplastin time (TPT), partial thromboplastin time (PTT), and platelet (PLT). *p* < 0.05: statistically significant.

Correlation Analysis	Pearson *r*	*p*-Value	Number of Pairs
**TAFI and C5a**	−0.4933	0.0104	26
**TAFI and leukocytes**	−0.2442	0.1855	31
**TAFI and lactate**	−0.4268	0.0423	23
**TAFI and pH**	0.2079	0.3532	22
**TAFI and heart rate**	−0.2603	0.2304	23
**TAFI and respiratory rate**	−0.3685	0.0915	22
**TAFI and SOFA score**	−0.3865	0.0422	28
**TAFI and INR**	−0.0499	0.7860	32
**TAFI and TPT**	0.0299	0.8750	30
**TAFI and PTT**	0.0369	0.8409	32
**TAFI and PLT**	−0.3048	0.0085	33
**C5a and leukocytes**	−0.2260	0.1918	35
**C5a and lactate**	0.4542	0.0173	27
**C5a and pH**	−0.0805	0.6839	28
**C5a and heart rate**	0.2416	0.2068	29
**C5a and respiratory rate**	0.4185	0.0266	28
**C5a and SOFA score**	0.3795	0.0386	30
**C5a and INR**	−0.0199	0.9055	38
**C5a and TPT**	−0.2056	0.2291	36
**C5a and PTT**	0.0731	0.6629	38
**C5a and PLT**	0.0138	0.9353	37

**Table 5:** Receiver-operator curve (ROC) analyses on TAFI, C5a, leukocyte count, lactate level, heart rate, and respiratory rate at admission to the emergency department to predict the development of sepsis after polytrauma. Sensitivity and specificity are given as percentage values. Abbreviations: AUC, area under the curve and CI, confidence interval.

Parameter	Cut-Off Value	Sensitivity % (95% CI)	Specificity % (95% CI)	AUC (95% CI)	*p*-Value
**TAFI [ng/mL]**	>54857	64.00 (42.52 to 82.03)	87.50 (47.35 to 99.68)	0.7550 (0.585 to 0.925)	0.032
**C5a [ng/mL]**	<17.00	75.00 (55.13 to 89.31)	70.00 (34.75 to 93.33)	0.7286 (0.547 to 0.910)	0.034
**leukocytes [U/nL]**	<14.50	65.63 (46.81 to 81.43)	69.23 (38.57 to 90.91)	0.7043 (0.527 to 0.882)	0.033
**lactate [mg/dL]**	<28.50	78.26 (56.30 to 92.54)	69.23 (38.57 to 90.91)	0.6973 (0.500 to 0.895)	0.052
**heart rate**	<102.5	86.96 (66.41 to 97.22)	64.29 (35.14 to 87.24)	0.6957 (0.490 to 0.902)	0.049
**respiratory rate**	<15.00	63.64 (40.66 to 82.80)	64.29 (35.14 to 87.24)	0.7029 (0.511 to 0.895)	0.043
